# Supplementary material for: Feature Extraction and Reconstruction by Using 2D-VMD Based on Carrier-Free UWB Radar Application in Human Motion Recognition
Source: Sensors (Basel). 2019 Apr 26;19(9):1962. doi: 10.3390/s19091962 (PMC6539238; doi:10.3390/s19091962)
Supplement: Supplementary file 1 [file sensors-19-01962-s001.pdf]

Appendix A

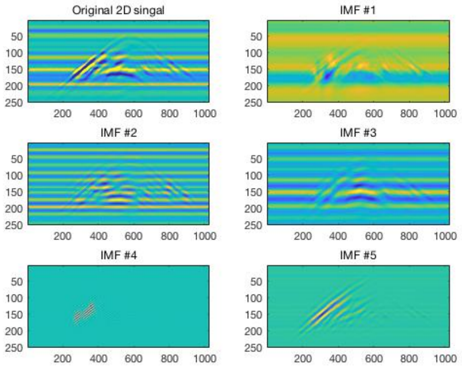

(a) Walk forward

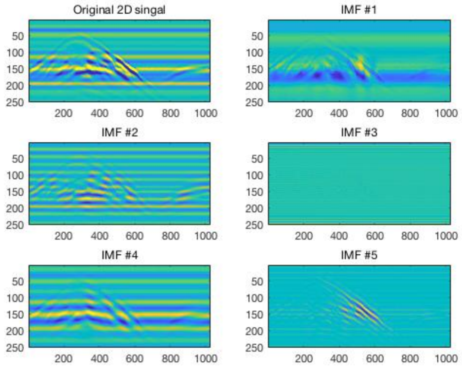

(b) Walk backward

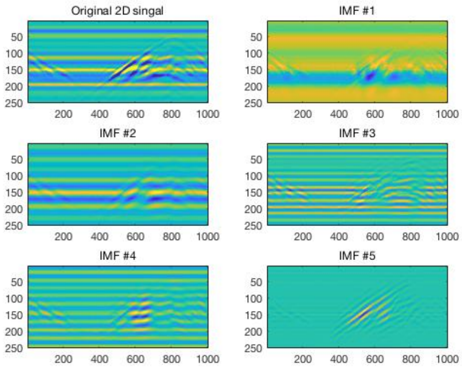

(c) Run forward

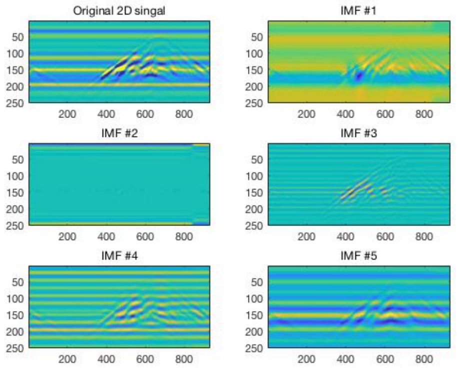

(d) Run backward

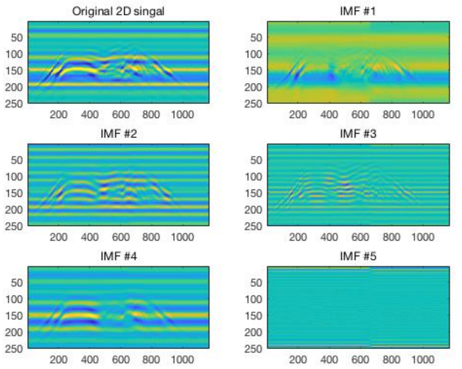

(e) Fall forward

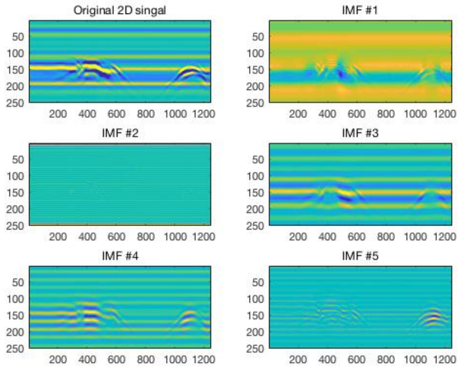

(f) Fall backward

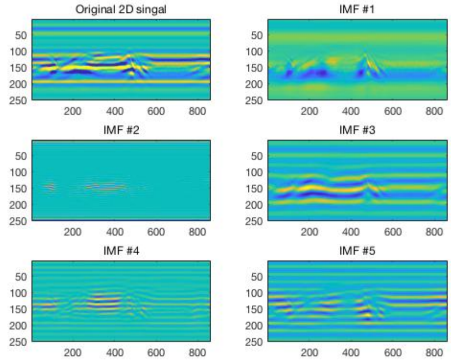

(g) Walk around

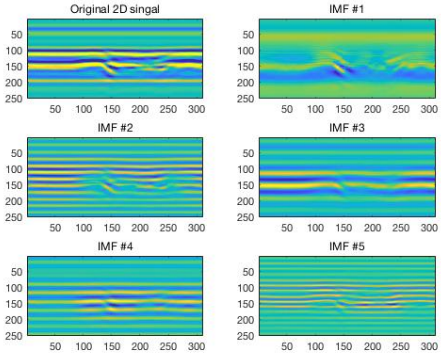

(h) Jump up and down

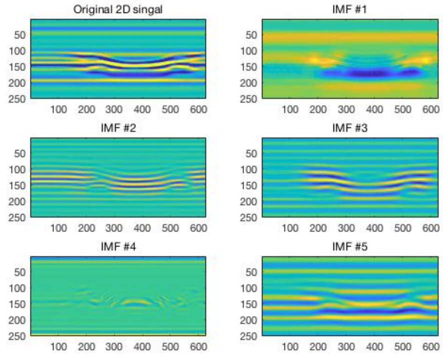

(i) Jump forward

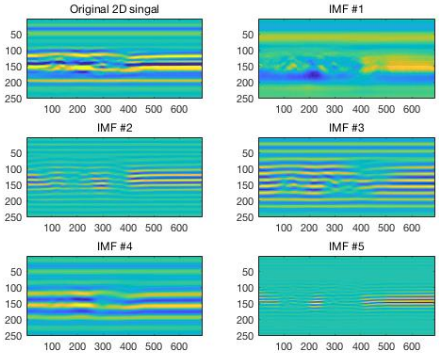

(j) Jump backward

Figure S1. Ten different types of human motion 2D echo signals and several BIMFs components after 2D-VMD decomposition. (a) walk forward of human motion 2D echo signal and several BIMFs component; (b) walk backward of human motion 2D echo signal and several BIMFs component; (c) run forward of human motion 2D echo signal and several BIMFs component; (d) run backward of human motion 2D echo signal and several BIMFs component; (e) fall forward of human motion 2D echo signal and several BIMFs component; (f) fall backward of human motion 2D echo signal and several BIMFs component; (g) walk around of human motion 2D echo signal and several BIMFs component; (h) jump up and down of human motion 2D echo signal and several BIMFs component; (i) jump forward of human motion 2D echo signal and several BIMFs component; (j) jump backward of human motion 2D echo signal and several BIMFs component.

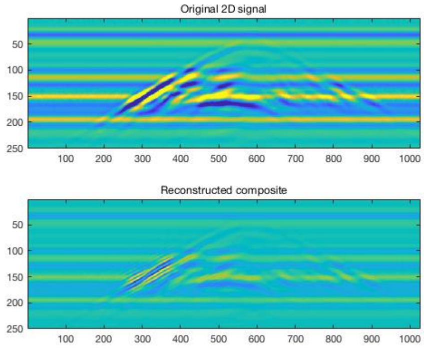

(a) Walk forward

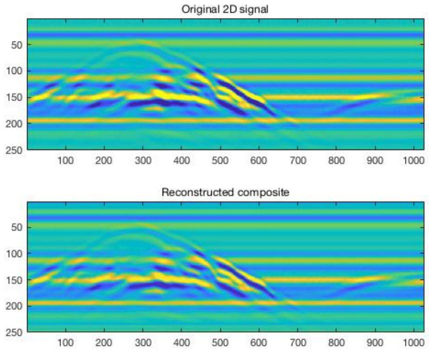

(b) Walk backward

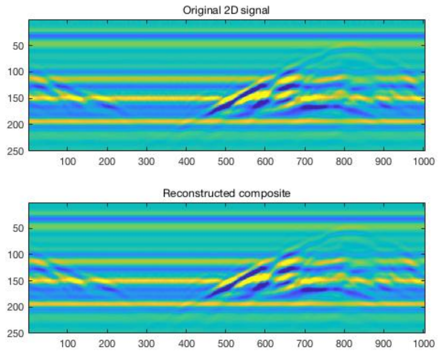

(c) Run forward

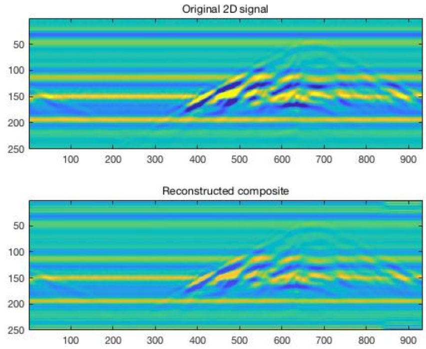

(d) Run backward

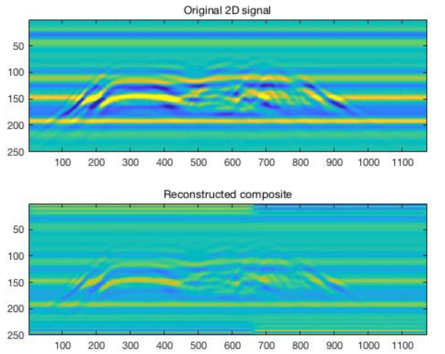

(e) Fall forward

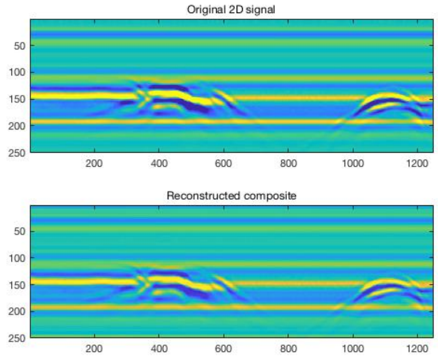

(f) Fall backward

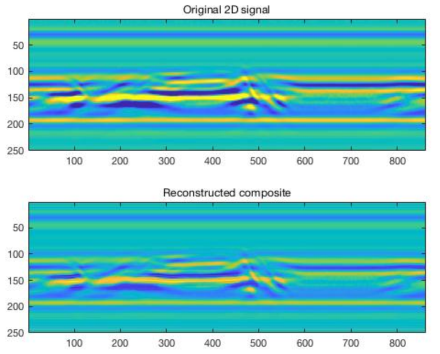

(g) Walk around

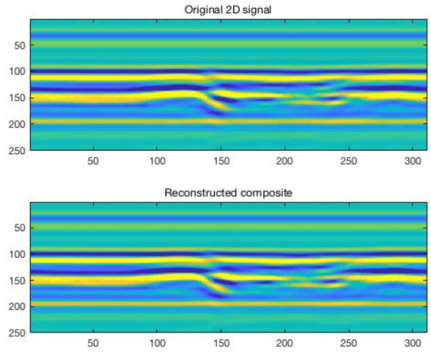

(h) Jump up and down

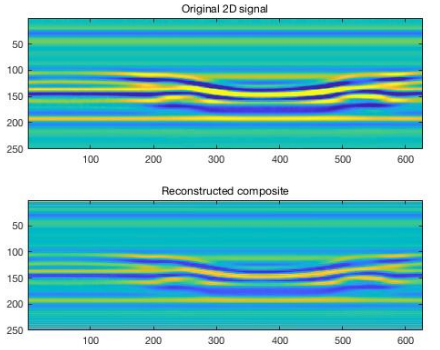

(i) Jump forward

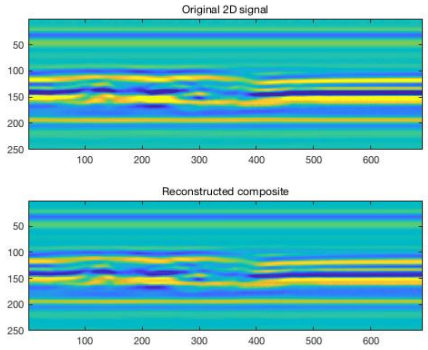

(j) Jump backward

Figure S2. Ten different types of human motion original 2D echo signals and reconstructed 2D echo signals (The above figure shows the original human motion radar 2D echo signal, the blow figure shows the reconstructed 2D radar echo signal). (a) walk forward of human motion original 2D echo signal and reconstructed 2D echo signal; (b) walk backward of human motion original 2D echo signal and reconstructed 2D echo signal; (c) run forward of human motion original 2D echo signal and reconstructed 2D echo signal; (d) run backward of human motion original 2D echo signal and reconstructed 2D echo signal; (e) fall forward of human motion original 2D echo signal and reconstructed 2D echo signal; (f) fall backward of human motion original 2D echo signal and reconstructed 2D echo signal; (g) walk around of human motion original 2D echo signal and reconstructed 2D echo signal; (h) jump up and down of human motion original 2D echo signal and reconstructed 2D echo signal; (i) jump forward of human motion original 2D echo signal and reconstructed 2D echo signal; (j) jump backward of human motion original 2D echo signal and reconstructed 2D echo signal.

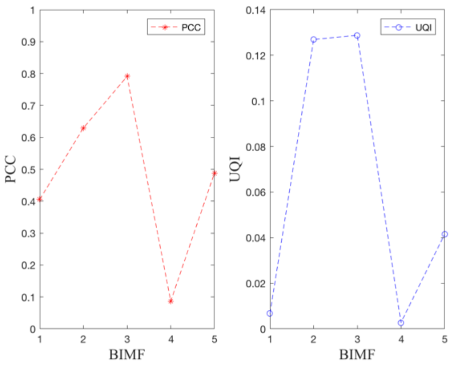

(a) Walk forward

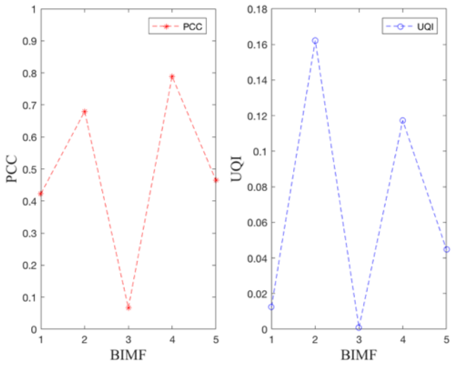

(b) Walk backward

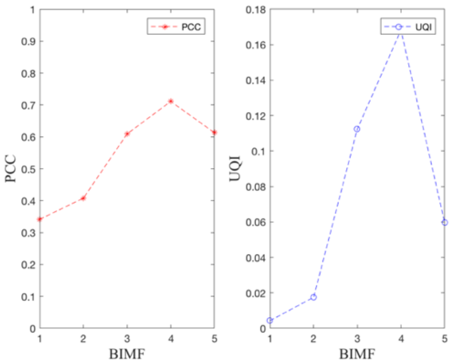

(c) Run forward

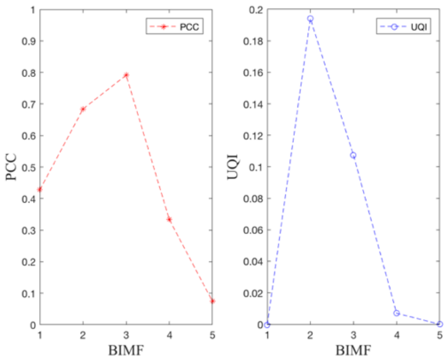

(d) Run backward

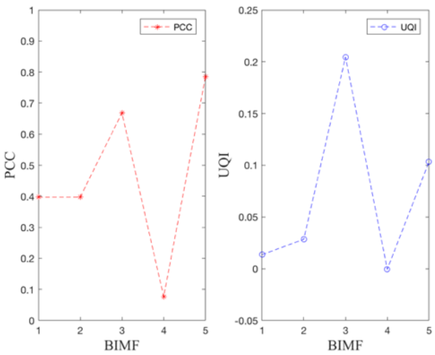

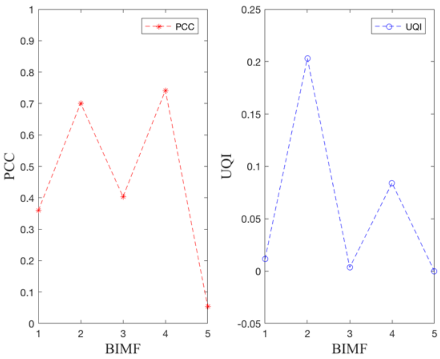

(e) Fall forward (f) Fall backward

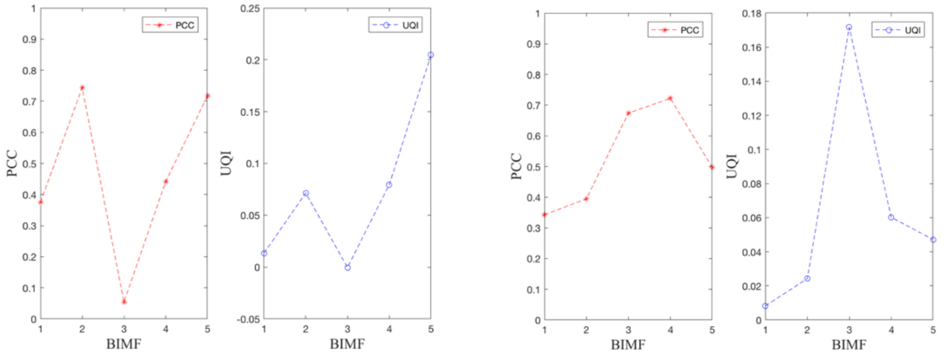

(g) Walk around (h) Jump up and down

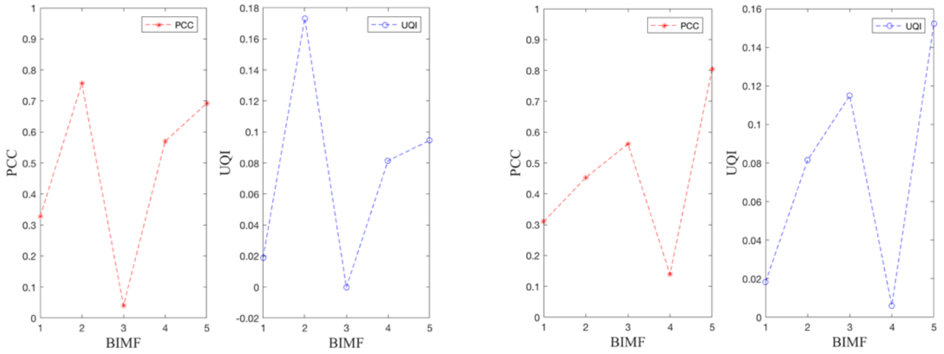

(i) Jump forward (j) Jump backward

Figure S3. PCC and UQI indicators for feature extraction of human motion 2D echo analysis signals of UWB radar. (a) The PCC and UQI indicators for feature extraction of walk forward of human motion 2D echo analysis signal; (b) The PCC and UQI indicators for feature extraction of walk backward of human motion 2D echo analysis signal; (c) The PCC and UQI indicators for feature extraction of run forward of human motion 2D echo analysis signal; (d) The PCC and UQI indicators for feature extraction of run backward of human motion 2D echo analysis signal; (e) The PCC and UQI indicators for feature extraction of fall forward of human motion 2D echo analysis signal; (f) The PCC and UQI indicators for feature extraction of fall backward of human motion 2D echo analysis signal; (g) The PCC and indicators for feature extraction of walk around of human motion 2D echo analysis signal; (h) The PCC and UQI indicators for feature extraction of jump up and down of human motion 2D echo analysis signal; (i) The PCC and UQI indicators for feature extraction of jump forward of human motion 2D echo analysis signal; (j) The PCC and UQI indicators for feature extraction of jump backward of human motion 2D echo analysis signal;

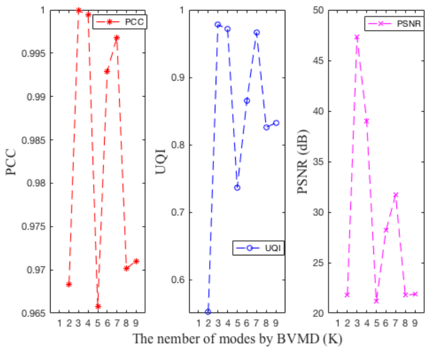

(a) Walk forward

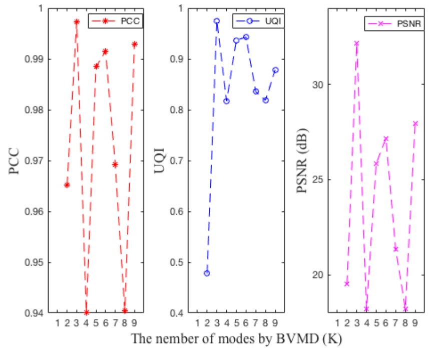

(b) Walk backward

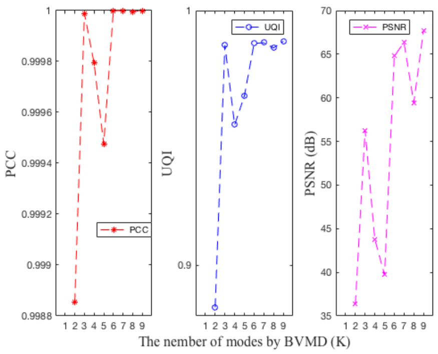

(c) Run forward

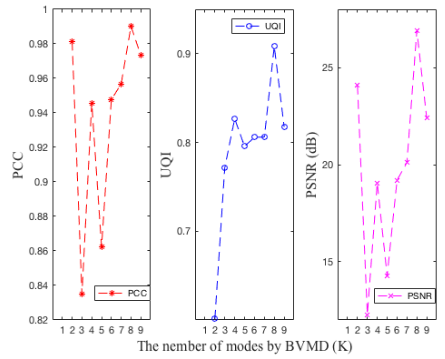

(d) Run backward

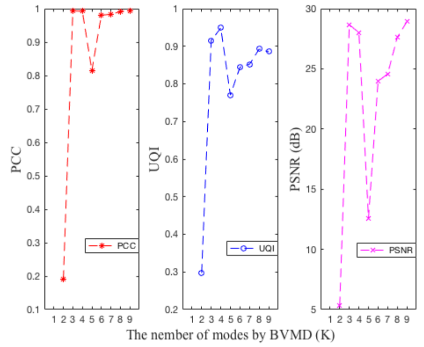

(e) Fall forward

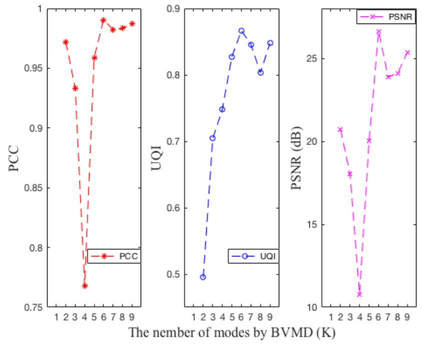

(f) Fall backward

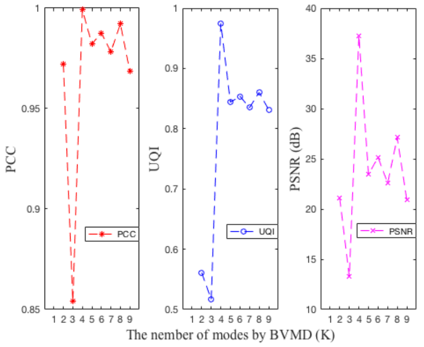

(g) Walk around

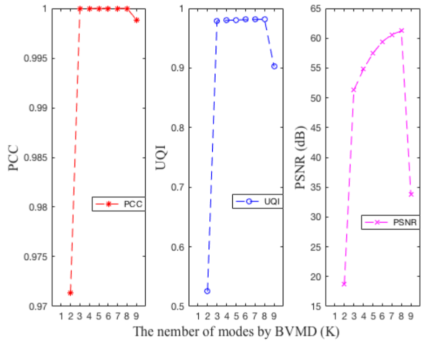

(h) Jump up and down

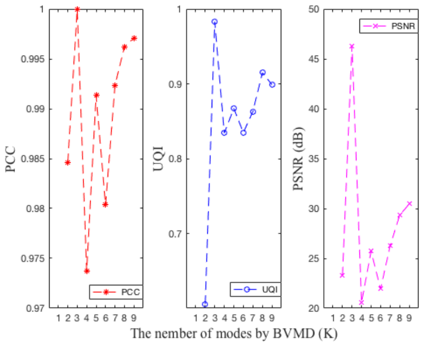

(i) Jump forward

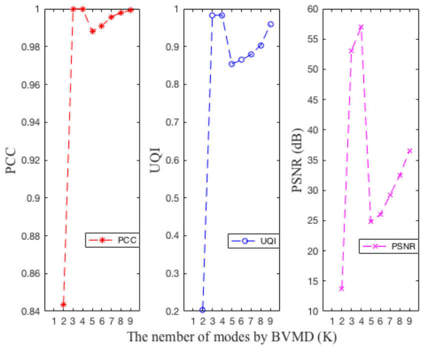

(j) Jump backward

Figure S4. Three indicators of PCC, UQI and PSNR of 2D echo signal reconstruction of ten different types of UWB radar-based human motion 2D echo analysis signal. (a) The three indicators of PCC, UQI and PSNR of 2D echo signal reconstruction of walk forward of UWB radar-based human motion 2D echo analysis signal; (b) The three indicators of PCC, UQI and PSNR of 2D echo signal reconstruction of walk backward of UWB radar-based human motion 2D echo analysis signal; (c) The three indicators of PCC, UQI and PSNR of 2D echo signal reconstruction of run forward of UWB radar-based human motion 2D echo analysis signal; (d) The three indicators of PCC, UQI and PSNR of 2D echo signal reconstruction of run backward of UWB radar-based human motion 2D echo analysis signal; (e) The three indicators of PCC, UQI and PSNR of 2D echo signal reconstruction of fall forward of UWB radar-based human motion 2D echo analysis signal; (f) The three indicators of PCC, UQI and PSNR of 2D echo signal reconstruction of fall backward of UWB radar-based human motion 2D echo analysis signal; (g) The three indicators of PCC, UQI and PSNR of 2D echo signal reconstruction of walk around of UWB radar-based human motion 2D echo analysis signal; (h) The three indicators of PCC, UQI and PSNR of 2D echo signal reconstruction of jump up and down of UWB radar-based human motion 2D echo analysis signal; (i) The three indicators of PCC, UQI and PSNR of 2D echo signal reconstruction of jump forward of UWB radar-based human motion 2D echo analysis signal; (j) The three indicators of PCC, UQI and PSNR of 2D echo signal reconstruction of jump backward of UWB radar-based human motion 2D echo analysis signal.

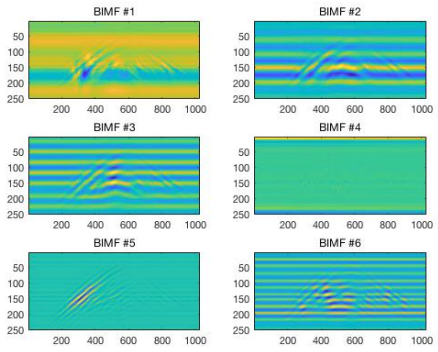

(a) Walk forward

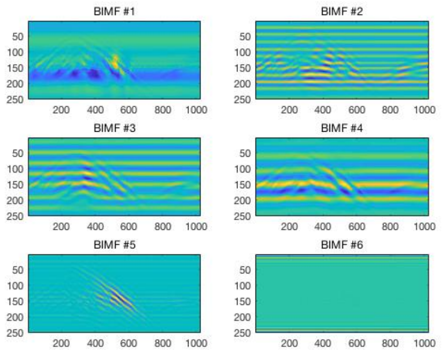

(b) Walk backward

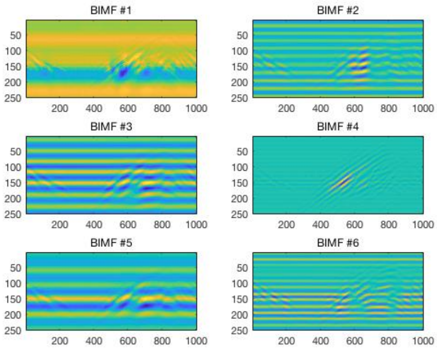

(c) Run forward

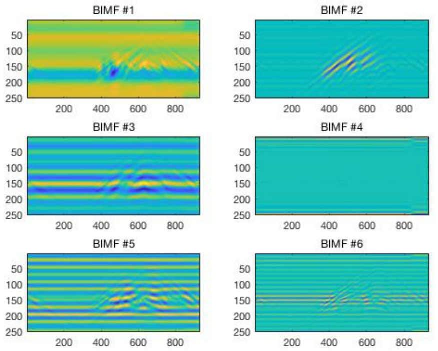

(d) Run backward

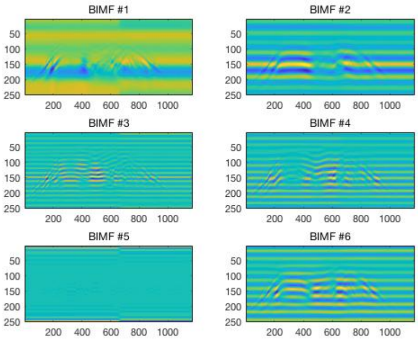

(e) Fall forward

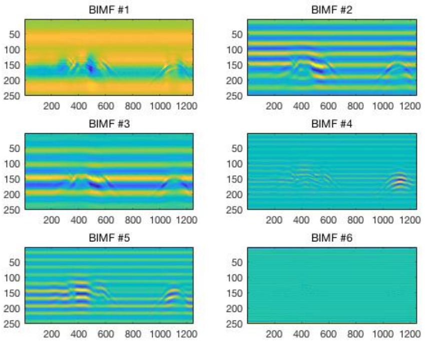

(f) Fall backward

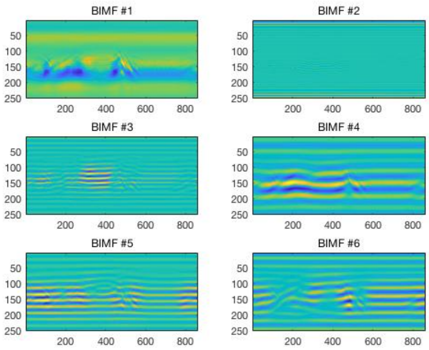

(g) Walk around

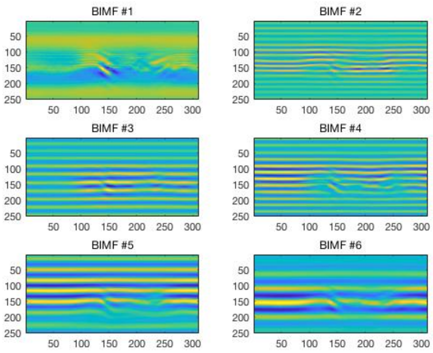

(h) Jump up and down

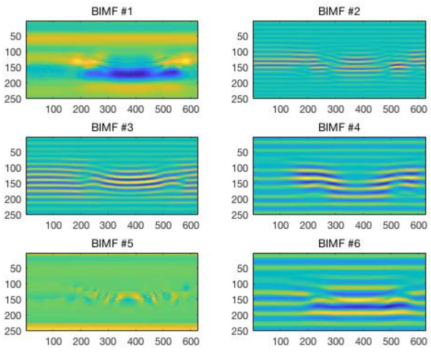

(i) Jump forward

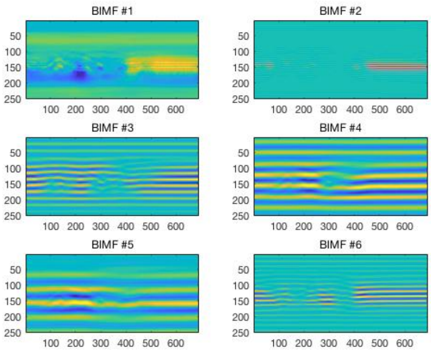

(j) Jump backward

Figure S5. The six BIMF mode components of feature extraction of the carrier-free UWB radar human motion 2D echo analysis signal when the 2D-VMD decomposition layer number is six. (a) The six BIMF mode components of feature extraction of walk forward of the carrier-free UWB radar human motion 2D echo analysis signal; (b) The six BIMF mode components of feature extraction of walk backward of the carrier-free UWB radar human motion 2D echo analysis signal; (c) The six BIMF mode components of feature extraction of run forward of the carrier-free UWB radar human motion 2D echo analysis signal; (d) The six BIMF mode components of feature extraction of run backward of the carrier-free UWB radar human motion 2D echo analysis signal; (e) The six BIMF mode components of feature extraction of fall forward of the carrier-free UWB radar human motion 2D echo analysis signal; (f) The six BIMF mode components of feature extraction of fall backward of the carrier-free UWB radar human motion 2D echo analysis signal; (g) The six BIMF mode components of feature extraction of walk around of the carrier-free UWB radar human motion 2D echo analysis signal; (h) The six BIMF mode components of feature extraction of jump up and down of the carrier-free UWB radar human motion 2D echo analysis signal; (i) The six BIMF mode components of feature extraction of the carrier-free UWB radar human motion 2D echo analysis signal; (j) The six BIMF mode components of feature extraction of jump backward of the carrier-free UWB radar human motion 2D echo analysis signal.

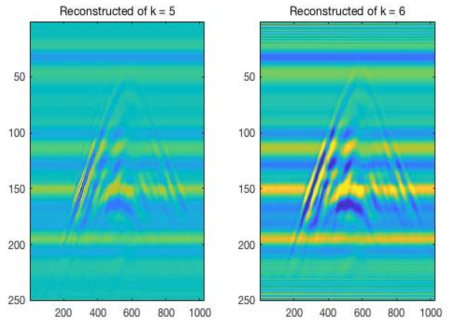

(a) Walk forward

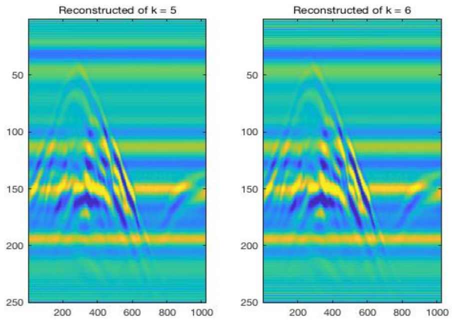

(b) Walk backward

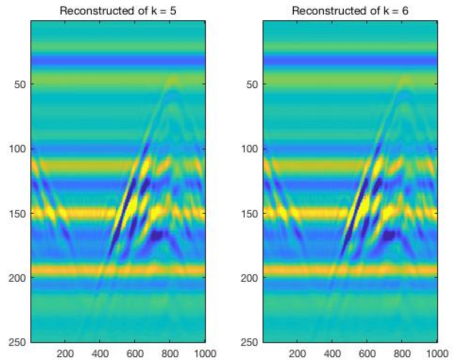

(c) Run forward

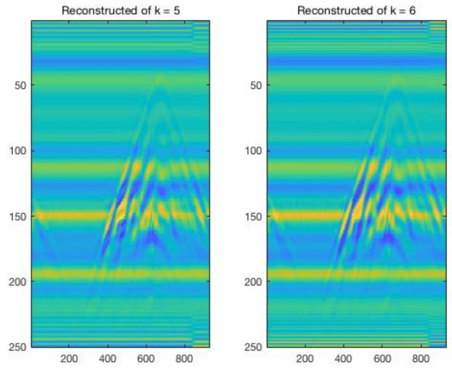

(d) Run backward

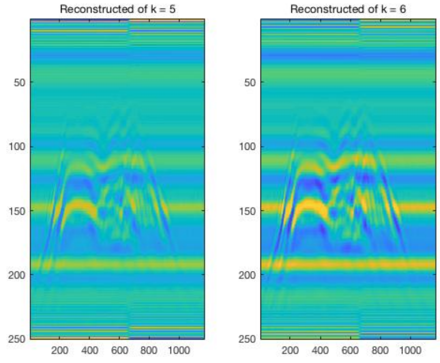

(e) Fall forward

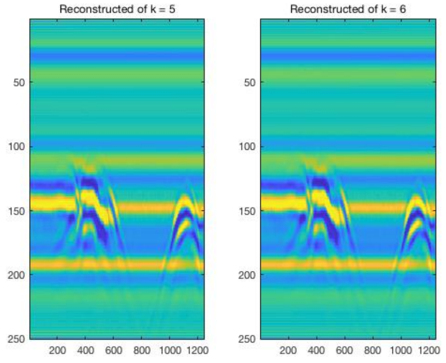

(f) Fall backward

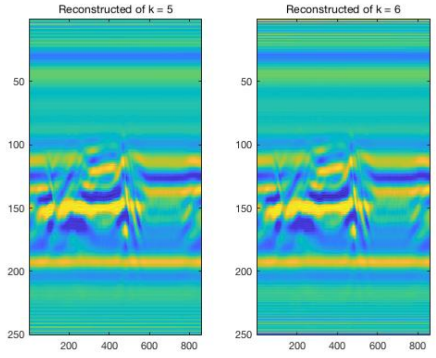

(g) Walk around

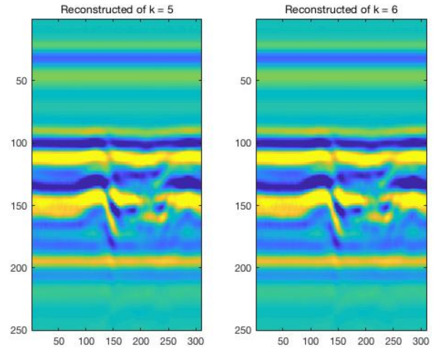

(h) Jump up and down

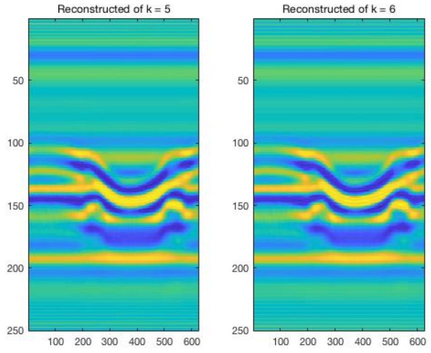

(i) Jump forward

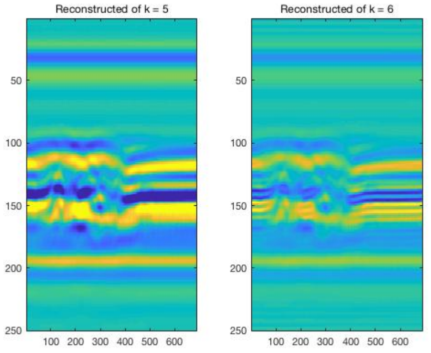

(j) Jump backward

Figure S6. Reconstruction of 2D human motion echo analysis signals of ten different types of carrier-free UWB radar. (The figure on the left shows the reconstruction of 2D echo signal of human motion of the carrier-free UWB radar through 2D-VMD algorithm when k=5, and the figure on the right shows the reconstruction of 2D echo signal of human motion of carrier-free UWB radar through 2D-VMD algorithm when k=6) (a) Reconstruction of 2D human motion echo analysis signal of walk forward of carrier-free UWB radar; (b) Reconstruction of 2D human motion echo analysis signal of walk backward of carrier-free UWB radar; (c) Reconstruction of 2D human motion echo analysis signal of run forward of carrier-free UWB radar; (d) Reconstruction of 2D human motion echo analysis signal of run backward of carrier-free UWB radar; (e) Reconstruction of 2D human motion echo analysis signal of fall forward of carrier-free UWB radar; (f) Reconstruction of 2D human motion echo analysis signal of fall backward of carrier-free UWB radar; (g) Reconstruction of 2D human motion echo analysis of walk around of carrier-free UWB

radar; (h) Reconstruction of 2D human motion echo analysis signal of jump up and down of carrier-free UWB radar; (i) Reconstruction of 2D human motion echo analysis signal; (j) Reconstruction of 2D human motion echo analysis signal of jump backward of carrier-free UWB radar.

Appendix B

2. Two-dimensional variational mode decomposition (2D-VMD) algorithm

Dragomiretskiy and Zosso extended the VMD method to two-dimensional VMD(2D-VMD), which can adaptively decompose an 2D analysis signal into several different modes of separate spectral bands which have specific directional and oscillatory characteristics [23]. 2D-VMD leads to band-limited intrinsic mode functions (BLIMFs, 2D-BLIMFs for the bi-dimensional case). Each mode has a limited spectral bandwidth: the first BLIMF contains the highest frequencies and the last one contains the lowest frequencies. More precisely, VMD/2D-VMD consists in searching a specific number of modes and their respective center frequencies used to reproduce the original signal/image either exactly or in least-square sense. The modes are extracted concurrently. In the one-dimensional case (1D signal), VMD decomposes the signal into K discrete modes (BLIMFs). Each mode has a limited spectral bandwidth [18]: each mode is compact around a center pulsation determined during the decomposition.

2.1. 2D analysis signal

The literature [26] clarifies that the 1D analysis signal is realized by suppressing the negative frequency, so the 2D analysis signal must set a half plane of the frequency domain to zero. In this paper, a unit vector $\hat{e}=\left(\cos\left(\theta\right),\sin\left(\theta\right)\right)$ is introduced as the reference direction. When $\hat{e}\cdot u>0$, where u is a frequency signal, it is called positive frequency, and when $\hat{e}\cdot u<0$, it is called negative frequency. In summary, a 2D analysis signal is defined as follows:

Def 1. If f is a 2D signal and F is the Fourier transform of f. The Fourier transform of the 2D analysis signal can be defined as:

$$F_A\left(\vec{\omega}\right)=\begin{cases}2F\left(\omega\right) & if\, \omega\cdot\hat{e}>0\\ F\left(\omega\right) & if\, \omega\cdot\hat{e}=0\\ 0 & if\, \omega\cdot\hat{e}<0\end{cases} \quad (1)$$

$$=F\left(\omega\right)\left(1+\operatorname{sgn}\left(\omega\cdot\hat{e}\right)\right)$$

According to the defined in the literature [26], the spectral domain of the 2D signal is expressed as:

$$f_A\left(\vec{x}\right)=f\left(\vec{x}\right)**\left(\delta\left(\vec{x}\cdot\vec{\omega}_k\right)+\frac{j}{\pi\left(\vec{x}\cdot\vec{\omega}_k\right)}\right)\delta\left(\vec{x}\cdot\vec{\omega}_{k,\perp}\right) \quad (2)$$

Where $**$ represents a 2D convolution, $\vec{\omega}_{k,\perp}$ represents a unit vector and $\vec{\omega}\cdot\vec{\omega}_{k,\perp}=0$.

According to the defined of the above formula, the 2D analysis signal is calculated line by line along the reference direction, and each calculation is processed independently.

2.2. Two-dimensional variational modal function model

The core of the 2D-VMD decomposition algorithm is to decompose a 2D analysis signal to be detected into a number of 2D intrinsic mode components $\hat{u}_k\left(\vec{x}\right)$, which can be calculated by the following steps:

(1) The 2D signal $\hat{u}_{AS,k}(\vec{\omega})$ is subjected to a 2D-Hilbert transform to obtain an analytical signal of 2D signal $\hat{u}_{AS,k}(\vec{\omega})$, thereby calculating a signal-sided 2D spectrum of $\hat{u}_{AS,k}(\vec{\omega})$.

$$\hat{u}_{AS,k}(\vec{x}) = \hat{u}_k(\vec{x}) ** \left(\delta\left(\left\langle \vec{x}, \vec{\omega}_k \right\rangle\right) + \frac{j}{\pi \left\langle \vec{x}, \vec{\omega}_k \right\rangle} \right) \delta\left(\left\langle \vec{x}, \vec{\omega}_{k,\perp} \right\rangle\right) \tag{3}$$

Where $\delta(t)$ is the unit impulse function; j is the imaginary unit; $*$ is the convolution.

(2) The parsed signal of the intrinsic mode component $\hat{u}_{AS,k}(\vec{\omega})$ is estimated for its center frequency $e^{-j\left\langle \vec{\omega}, \vec{\omega}_k \right\rangle}$, and then the spectrum of each $\hat{u}_{AS,k}(\vec{\omega})$ is modulated onto its corresponding frequency baseband.

$$\left[\hat{u}_k(\vec{x}) * \left(\delta\left(\left\langle \vec{x}, \vec{\omega}_k \right\rangle\right) + \frac{j}{\pi \left\langle \vec{x}, \vec{\omega}_k \right\rangle} \right) \delta\left(\left\langle \vec{x}, \vec{\omega}_{k,\perp} \right\rangle\right) \right] e^{-j\left\langle \vec{x}, \vec{\omega}_k \right\rangle} \tag{4}$$

(3) Finally, calculate the square of the demodulated signal gradient L_2 norm in step (2).

$$\left\| \nabla \left[\hat{u}_k(\vec{x}) * \left(\delta\left(\left\langle \vec{x}, \vec{\omega}_k \right\rangle\right) + \frac{j}{\pi \left\langle \vec{x}, \vec{\omega}_k \right\rangle} \right) \delta\left(\left\langle \vec{x}, \vec{\omega}_{k,\perp} \right\rangle\right) \right] e^{-j\left\langle \vec{x}, \vec{\omega}_k \right\rangle} \right\|_2^2 \tag{5}$$

Where ∇ represents the second derivative.

The variational constraint model of the 2D analysis signal obtained by the above steps (1) ~ (3).

$$\min_{\left\{\vec{u}_k, \vec{\omega}_k\right\}} \left\{ \sum_{k=1}^{K} \left\| \nabla \left[\hat{u}_k(\vec{x}) * \left(\delta\left(\left\langle \vec{x}, \vec{\omega}_k \right\rangle\right) + \frac{j}{\pi \left\langle \vec{x}, \vec{\omega}_k \right\rangle} \right) \delta\left(\left\langle \vec{x}, \vec{\omega}_{k,\perp} \right\rangle\right) \right] e^{-j\left\langle \vec{x}, \vec{\omega}_k \right\rangle} \right\|_2^2 \right\} \tag{6}$$

$$s.t. \ \sum_{k=1}^{K} \hat{u}_k(x) = f$$

Where $\hat{u}_k = \left\{u_1, u_2, ..., u_k\right\}$ represents the set of each modes after 2D mode decomposition; $\omega_k = \left\{\omega_1, \omega_2, ..., \omega_k\right\}$ represents the set of center frequencies corresponding

to each mode after the 2D variational mode decomposition.

The above 2D-VMD constraint model is actually a two-dimensional (2D) constrained optimal solution problem. In order to solve the above optimization problem, this paper uses Lagrange multiplication with the augmented Lagrange matrix function L to solve the optimal solution.

2.3. Solution of two-dimensional variational constraint model

The two-dimensional variational mode decomposition (2D-VMD) constraint model in Section 2.2. It is actually an optimal model of two-dimensional constraints. In this section, the augmented Lagrange matrix function L by using Lagrange number multiplication is introduced. it can be obtained by Equation (7).

$$L(\{u_k\},\{\omega_k\},\lambda) := \alpha\sum_{k=1}^{K}\left\|\nabla\left[\hat{u}_k(\vec{x}) * \left(\delta\left(\langle\vec{x},\vec{\omega}_k\rangle\right) + \frac{j}{\pi\langle\vec{x},\vec{\omega}_k\rangle}\right)\delta\left(\langle\vec{x},\vec{\omega}_{k,\perp}\rangle\right)\right]e^{-j\langle\vec{x},\vec{\omega}_k\rangle}\right\|_2^2 + \left\|f-\sum_{k=1}^{K}u_k(x)\right\|_2^2 + \left\langle\lambda(x), f-\sum_{k=1}^{K}u_k(x)\right\rangle \tag{7}$$

Where α represents the penalty factor parameter; λ is Lagrange multiplier.

The alternating direction multiplier algorithm is used to solve the optimal solution of the augmented Lagrange function L of the upper variational model.

Then, $\hat{u}_k^{n+1}$ can be expressed as:

$$\hat{u}_k^{n+1} = \arg\min_{\hat{u}_{k\in f}}\left\{\left\{\alpha\sum_{k=1}^{K}\left\|\nabla\left[\hat{u}_k(\vec{x}) * \left(\delta\left(\langle\vec{x},\vec{\omega}_k\rangle\right) + \frac{j}{\pi\langle\vec{x},\vec{\omega}_k\rangle}\right)\delta\left(\langle\vec{x},\vec{\omega}_{k,\perp}\rangle\right)\right]e^{-j\langle\vec{x},\vec{\omega}_k\rangle}\right\|_2^2 + \left\|f-\sum_{k=1}^{K}u_k(x)+\frac{\lambda(\mathrm{t})}{2}\right\|_2^2\right\} \tag{8}$$

According to the Parseval/Plancherel Fourier isometric transform, the above formula is transformed from the time domain to the frequency domain as:

$$\hat{u}_k^{n+1} = \arg\min_{\hat{u}_k,\hat{u}_{k\in f}}\left\{\alpha\left\|j\left(\vec{\omega}-\vec{\omega}_k\right)\left[\left(1+\operatorname{sgn}\left(\langle\vec{\omega},\vec{\omega}_k\rangle\right)\right)\hat{u}_k(\omega)\right]\right\|_2^2 + \left\|f\left(\vec{\omega}\right)-\sum_{k=1}^{K}u_k(\omega)+\frac{\lambda(t)}{2}\right\|_2^2\right\} \tag{9}$$

In the above formula, let $\omega = \omega - \omega_k$, then the above formula is converted to the Equation (10):

$$\hat{u}_k^{n+1} = \arg\min_{\hat{u}_k, \hat{u}_{k\in f}} \left\{ \alpha \left\| j\left(\vec{\omega} - \vec{\omega}_k\right)\left[\left(1 + \operatorname{sgn}\left(\left\langle \vec{\omega}, \vec{\omega}_k \right\rangle\right)\right)\hat{u}_k(\omega)\right] \right\|_2^2 + \left\| f\left(\vec{\omega}\right) - \sum_{k=1}^{K} u_k(\omega) + \frac{\lambda(t)}{2} \right\|_2^2 \right\} \tag{10}$$

According to the Hermitian symmetry characteristic of the original signal in the signal reconstruction fidelity term, the two terms in the above formula are converted into the form of the upper half space integral of the non-negative frequency.

$$\hat{u}_k^{n+1} = \arg\min_{\hat{u}_k, \hat{u}_{k\in f}} \left\{ \int_0^\infty \left[4\alpha\left(\omega - \omega_k\right)^2 \left|\hat{u}_k(\omega)\right|^2 + 2\left| f\left(\vec{\omega}\right) - \sum_{k=1}^{K} u_k(\omega) + \frac{\lambda(t)}{2} \right|^2 \right] d\omega \right\} \tag{11}$$

Finally, the solution of two-dimensional variational constraint model also conforms to the form of wiener filter. The specific expression is as follows:

$$\hat{u}_k^{n+1}\left(\vec{\omega}\right) = \frac{\hat{f}\left(\vec{\omega}\right) - \sum_{k=1}^{K} u_k(\omega) + \frac{\lambda(t)}{2}}{1 + 2\alpha\left(\omega - \omega_k\right)^2} \tag{12}$$

Similarly, the updated formula for the corresponding central frequency of each mode after two-dimensional variational mode decomposition can be obtained as follows:

$$\omega_k^{n+1} = \arg\min_{\vec{\omega}_k} \left\{ \sum_{k=1}^{K} \left\| \nabla \left[\left[\hat{u}_k(\vec{x}) * \left(\delta\left(\left\langle \vec{x}, \vec{\omega}_k \right\rangle\right) + \frac{j}{\pi \left\langle \vec{x}, \vec{\omega}_k \right\rangle} \right) \delta\left(\left\langle \vec{x}, \vec{\omega}_{k,\perp} \right\rangle\right) \right] e^{-j\left\langle \vec{x}, \vec{\omega}_k \right\rangle} \right] \right\|_2^2 \right\} \tag{13}$$

According to the Parseval/Plancherel Fourier isometric transform, the above formula is transformed from the time domain to the frequency domain as:

$$\omega_k^{n+1} = \arg\min_{\vec{\omega}_k} \left\{ \alpha \left\| j\left(\vec{\omega} - \vec{\omega}_k\right)\left[\left(1 + \operatorname{sgn}\left(\vec{\omega}, \vec{\omega}_k\right)\right)\hat{u}_k\left(\vec{\omega}\right)\right] \right\|_2^2 \right\} \tag{14}$$

Similarly, the solution to update center frequency ω_k^{n+1} is:

$$\vec{\omega}_k^{n+1} = \frac{\int_{\Omega_k} \vec{\omega} \left|\hat{u}_k\left(\vec{\omega}\right)\right|^2 d\vec{\omega}}{\int_{\Omega_k} \left|\hat{u}_k\left(\vec{\omega}\right)\right|^2 d\vec{\omega}} \tag{15}$$

Where the iteration step criterion is Equation (16).

$$\sum_{k=1}^{K}\frac{\left\|\hat{u}_k^{n+1}-\hat{u}_k^{n}\right\|_2^2}{\left\|\hat{u}_k^{n}\right\|_2^2}<\varepsilon \tag{16}$$

The 2D-VMD algorithm simply updates each intrinsic mode component directly in frequency until the iteration stop condition is satisfied, and then output each intrinsic mode component.

The 2D-VMD algorithm is a result obtained by continuously updating the 2D analysis signal in the frequency and then performing inverse Fourier transform. The specific process of the 2D-VMD algorithm is described as follows:

a) Initialize $\left\{\hat{u}_k^{1}\right\}$ 、 $\left\{\hat{\omega}_k^{1}\right\}$ 、 $\left\{\hat{\lambda}^{1}\right\}$ and n；

b) Update u_k and ω_k in frequency according to the above formula;

c) Update λ, where

$$\hat{\lambda}^{n+1}(\omega)=\hat{\lambda}^{n}(\omega)+\tau\left(\hat{f}(\omega)-\sum_{k=1}^{K}\hat{u}_k^{n+1}(\omega)\right) \tag{17}$$

d) Until $\sum_{k=1}^{K}\frac{\left\|\hat{u}_k^{n+1}-\hat{u}_k^{n}\right\|_2^2}{\left\|\hat{u}_k^{n}\right\|_2^2}<\varepsilon$, Stop iteration.

Algorithm 2D-VMD
Input: signal $f(x)$, number of modes k, parameters α_k, τ, ϵ.
Output: modes $u_k(x)$, center frequencies ω_k.
Initialize $\{\omega_k^0\}, \{\hat{u}_k^0\} \leftarrow 0, \hat{\lambda}^0 \leftarrow 0, n \leftarrow 0$
repeat
$n \leftarrow n+1$
for k = 1 : K do
Create 2D mask for analytic signal Fourier multiplier:
$\mathcal{H}_k^{t+1}(\omega) \leftarrow 1+sgn(\langle\omega_k^t,\omega\rangle)$
Update $\hat{u}_{AS,k}$:
$\hat{u}_{AS,k}^{t+1}(\omega) \leftarrow \mathcal{H}_k^{t+1}(\omega)\left[\frac{\hat{f}(\omega)-\sum_{i<k}\hat{u}_i^{t+1}(\omega)-\sum_{i>k}\hat{u}_i^{t}(\omega)+\frac{\hat{\lambda}^t(\omega)}{2}}{1+2\alpha_k\lvert\omega-\omega_k^t\rvert^2}\right]$ (18)
Update ω_k:

$$\omega_k^{t+1} \leftarrow \frac{\int_{\mathbb{R}^2} \omega \left|\hat{u}_{AS,k}^{t+1}(\omega)\right|^2 d\omega}{\int_{\mathbb{R}^2} \left|\hat{u}_{AS,k}^{t+1}(\omega)\right|^2 d\omega} \tag{19}$$

Retrieve u_k:

$$u_k^{t+1}(x) \leftarrow \mathcal{R}\left(\mathcal{F}^{-1}\{\hat{u}_{AS,k}^{t+1}(\omega)\}\right) \tag{20}$$

end for

Dual ascent (optional):

$$\hat{\lambda}^{t+1}(\omega) \leftarrow \hat{\lambda}^{t}(\omega) + \tau\left(\hat{f}(\omega) - \sum_k \hat{u}_k^{t+1}(\omega)\right) \tag{21}$$

Until convergence: $\sum_k \frac{\left\|\hat{u}_k^{t+1} - \hat{u}_k^{t}\right\|_2^2}{\left\|\hat{u}_k^{t}\right\|_2^2} < \epsilon.$ (22)
